# MARINE AND COASTAL SCIENCE: Will Ocean Acidification Erode the Base of the Food Web?

**DOI:** 10.1289/ehp.118-a157

**Published:** 2010-04

**Authors:** Carol Potera

**Affiliations:** **Carol Potera**, based in Montana, has written for *EHP* since 1996. She also writes for *Microbe*, *Genetic Engineering News*, and the *American Journal of Nursing*

Acidification of the world’s oceans is already damaging coral reefs and could produce other unexpected chemical and biological consequences. Princeton University researchers now report that at low pH, phytoplankton take up less iron, a key nutrient needed for photosynthesis and growth. The results, reported in the 5 February 2010 issue of *Science*, suggest ocean acidification could have a profound impact on these tiny one-celled plants, which reside at the bottom of the food web and support commercially important fisheries.

Seawater becomes more acidic when atmospheric carbon dioxide (CO_2_) absorbed by the water is converted into carbonic acid. The acidity of oceans is changing very rapidly. The hydrogen ion concentration of surface ocean water (a reflection of pH) is now about 30% higher than it was 200 years ago, according to William Sunda, a research chemist at the National Oceanic and Atmospheric Association in Beaufort, North Carolina, while atmospheric concentrations of CO_2_ have risen by about 38%. Most of the research focus has been on how ocean acidification negatively impacts marine creatures, such as mollusks and corals, that form shells or exoskeletons from calcium carbonate [*EHP* 116:A292–A299 (2008)]. Little attention has been paid to how increasing acidity changes the chemistry and biological availability of essential nutrients such as iron.

In the current study, Dalin Shi, Francois M. M. Morel, and colleagues at Princeton University measured the uptake of iron in *Thalassiosira weissflogii*, *Thalassiosira oceanica*, *Phaeodactylum tricornutum*, and *Emiliana huxleyi*. As the researchers lowered the pH of model laboratory culture media from 8.6 to 7.7, they observed a significant decrease in the rate of iron uptake by all species. A similar trend occurred when laboratory phytoplankton were placed in natural seawater collected off the New Jersey coast and the open ocean near Bermuda. The average iron uptake rate decreased by 10–20% between the highest- and lowest-pH conditions in natural seawater. “The average pH of ocean water today is 8.08,” says Shi, a graduate student in oceanography.

Much of the iron in ocean water is strongly bound to natural organic chelators, such as siderophores, which bind and release iron in different ways. The research team examined the effect on iron uptake of 3 chemically different model chelators—the synthetic chelator ethylenediaminetetraacetic acid (EDTA) and two siderophores, desferriferrioxamine B (DFB) and azotochelin. As the pH dropped, iron availability was dramatically reduced by EDTA and moderately reduced by DFB, but was unchanged by azotochelin.

Little is known about how marine ligands bind and release iron in seawater. The model chelator findings “show in principle that lowering pH can decrease iron present for biological use, depending on the chelator. And the results with natural seawater show this also occurs with natural chelators,” says Sunda.

One conceivable consequence of limited iron due to ocean acidification could be a decline in phytoplankton populations, resulting in reduced fish harvests for human consumption, according to Morel, a professor of geosciences. “But this is all speculation,” he cautions. “The only thing we documented is a decrease in the bioavailability of dissolved iron in four laboratory organisms.”

Phytoplankton species perform almost all marine photosynthesis, a biochemical process that requires iron to convert CO_2_ from air into organic matter and oxygen. Some of this organic matter sinks, carrying carbon into the deep oceans; calculations by Josep D. Canadell and colleagues in the 20 November 2007 issue of *Proceedings of the National Academy of Sciences* estimate this “carbon pump” has absorbed about a quarter of the CO_2_ emitted by human activities. A decrease in iron availability through ocean acidification could restrict this carbon pump, resulting in an increase in atmospheric CO_2_, notes Sunda.

On the other hand, marine organisms may evolve their own nutritional coping strategies. For instance, Sunda and colleagues, recently discovered that members of the bacterial genus *Marinobacter*, which live in close contact with phytoplankton that cause harmful algal blooms, produce a novel siderophore that tightly binds iron in the dark. But when exposed to sunlight, the siderophore breaks down and releases an unbound form of iron that the phytoplankton readily take up to drive photosynthesis. The relationship is mutually beneficial; when the *Marinobacter* and phytoplankton species are grown separately, both grow poorly compared with when they grow together. “Nature is incredibly clever when it comes to obtaining scarce nutrients,” says Sunda, whose work was described in the 6 October 2009 issue of the *Proceedings of the National Academy of Sciences*.

## Figures and Tables

**Figure:**
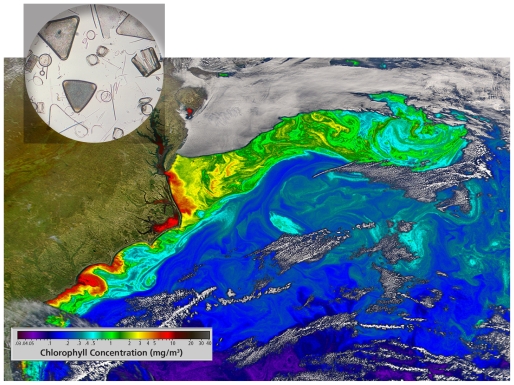
Ocean color remote sensing of chlorophyll concentrations off the U.S. East Coast reflects swirling fields of phytoplankton (inset). These microorganisms form the base of the marine food web.

